# Longitudinal changes in brain parenchyma due to mild traumatic brain injury during the first year after injury

**DOI:** 10.1002/brb3.2410

**Published:** 2021-10-28

**Authors:** Angela M. Muller, William J. Panenka, Rael T. Lange, Grant L. Iverson, Jeffrey R. Brubacher, Naznin Virji‐Babul

**Affiliations:** ^1^ Faculty of Medicine, Department of Physical Therapy, Djavad Mowafaghian Centre for Brain Health University of British Columbia Vancouver Canada; ^2^ British Columbia Neuropsychiatry Program University of British Columbia Vancouver Canada; ^3^ Department of Psychiatry University of British Columbia Vancouver Canada; ^4^ Defense and Veterans Brain Injury Center Walter Reed National Military Medical Center Bethesda Maryland USA; ^5^ National Intrepid Center of Excellence Walter Reed National Military Medical Center Bethesda Maryland USA; ^6^ Department of Physical Medicine and Rehabilitation Harvard Medical School Boston Massachusetts USA; ^7^ Department of Emergency Medicine University of British Columbia Vancouver Canada

**Keywords:** attention, concussion, gray matter volume, longitudinal, voxel‐wise morphometry, white matter

## Abstract

Chronic gray matter (GM) atrophy is a known consequence of moderate and severe traumatic brain injuries but has not been consistently shown in mild traumatic brain injury (mTBI). The aim of this study was to investigate the longitudinal effect of uncomplicated mTBI on the brain's GM and white matter (WM) from 6 weeks to 12 months after injury. Voxel‐based‐morphometry (VBM) was computed with the T1‐weighted images of 48 uncomplicated mTBI patients and 37 orthopedic controls. Over the period from 6 weeks to 12 months, only patients who experienced uncomplicated mTBI, but not control participants, showed significant GM decrease predominantly in the right hemisphere along the GM‐CSF border in lateral and medial portions of the sensorimotor cortex extending into the rolandic operculum, middle frontal gyrus, insula, and temporal pole. Additionally, only mTBI patients, but not controls, experienced significant WM decrease predominantly in the right hemisphere in the superior fasciculus longitudinalis, arcuate fasciculus, and cortical‐pontine tracts as well as a significant WM increase in left arcuate fasciculus and left capsula extrema. We did not observe any significant change in the controls for the same time interval or any significant group differences in GM and WM probability at each of the two timepoints. This suggests that the changes along the brain tissue borders observed in the mTBI group represent a reorganization associated with subtle microscopical changes in intracortical myelin and not a direct degenerative process as a result of mTBI.

## Abstract 2

One year after injury, mTBI patients showed GM and WM probability changes along the brain tissue borders indicating a reorganization. Given that the mTBI group improved in attention performance during the first year after injury, the reorganization seems rather to present subtle microscopical changes in intracortical myelin and not a direct degenerative process as a result of mTBI.

## 1 INTRODUCTION

In 2013, approximately 2.5 million emergency department visits were traumatic brain injury (TBI)‐related in the United States (Taylor et al., 2017). Approximately, 80% of these TBI patients are diagnosed with a mild traumatic brain injury (Glasgow Coma Scale 13–15) (Narayan et al., 2002). However, these numbers likely underestimate the true prevalence of mild traumatic brain injury (mTBI) as many patients with mTBI do not seek treatment or seek treatment in an out‐patient setting (Taylor et al., 2017). In the first few months following mTBI, symptoms such as headache, nausea, vertigo, poor sleep, depression, and cognitive difficulties are common (Williams et al., 2010. The majority of the mTBI patients recover to premorbid functioning within the first three months following injury, however, a substantial minority continue to experience symptoms beyond that time (Bigler et al., 2008; Iverson & Lange, 2011a, 2011b).

Diffuse white matter (WM) injury is a major pathology after mTBI (Omelchenko et al., 2019; Sharp et al., 2014). With diffusion tensor imaging (DTI), mTBI patients show lower fractional anisotropy and higher mean diffusivity values than healthy controls in the corpus callosum, the long WM projection and association fibers during the acute stage with only partial improvement during the chronic stage (Asken et al., 2018; Hellstrøm et al., 2017a; Lindsey et al., 2021). In addition to structural alterations, mTBI is also frequently associated with changed physiology and altered brain function. Studies using resting‐state fMRI to analyze the intrinsic connectivity networks of the brain have repeatedly shown that mTBI can result in altered synchronization and desynchronization patterns evidenced as alterations in correlation strength of the BOLD signal between different brain regions. The default mode network and the salience network have frequently been found to show impaired internetwork‐ as well as intranetwork activity following mTBI (Sharp et al., 2014).

Compared to the number of studies reporting changes in the WM architecture and the functional networks of the brain, the number of studies investigating the consequences of an mTBI on the gray matter (GM) is modest. Furthermore, the findings of studies reporting GM alterations after mTBI are difficult to compare because researchers use different GM parameters, that is, GM volume, GM thickness, GM surface, GM probability, and GM diffusion. How these different GM indicators are related with each other is still under debate (Panizzon et al., 2009; Winkler et al., 2010).

GM atrophy has consistently shown to be more pronounced in patients with moderate and severe TBI than in age‐matched healthy controls (Bendlin et al., 2008; Brezova et al., 2014; Cole et al., 2018; Cole et al., 2015; Ding et al., 2008; Gale et al., 2005; Ledig et al., 2017; Tomaiuolo et al., 2021; Tomaiuolo et al., 2012; Warner et al., 2010), but the findings for mTBI are mixed. The majority of the studies (among others (Dean et al., 2015; Govindarajan et al., 2016; Hellstrøm et al., 2017b; MacKenzie et al., 2002; Mayer et al., 2015; Sussman et al., 2017; Wang et al., 2015; Zhou et al., 2013)) document a decrease of GM in mTBI patients compared to healthy controls. In contrast, two studies (España et al., 2017; Ling et al., 2013) found no significant GM changes while two other studies document the reverse, namely an increase in GM volume (Killgore et al., 2016) and thickness (Dall'Acqua et al., 2017) 1 year after injury.

The primary aim of the present study was to use T1 weighted structural magnetic resonance imaging data to investigate the GM changes in mTBI patients from 6 weeks to 1‐year post injury in comparison with a control group of orthopedic patients. We used a voxel‐based longitudinal processing pipeline optimized to detect subtle changes in brain tissue in combination with a nonparametric statistic. Since voxel‐based morphometry (VBM) can also be used to analyze WM concentration in T1 weighted data, a secondary aim of the study was to investigate how the GM changes in the mTBI participants relate to concurrent changes in WM.

## 2 METHODS

### 2.1 Participants

Participants were drawn from a larger prospective inception cohort study of TBI patients presenting to Vancouver General Hospital (Adult level 1 trauma center). Patients were recruited to the parent study via daily reviews of admission to the emergency department between 2007 and 2014. They were enrolled in the study if they were (a) between 19 and 55 years of age, (b) injured as a result of a traumatic injury (e.g., fall, motor vehicle accident, assault, etc.), and (c) had a blood alcohol level (BAL) obtained at the time of injury by hospital staff on admission to the Emergency Department (to meet the parent study primary hypothesis about the interaction of alcohol and TBI). General exclusion criteria included (a) lack of proficiency in conversational English; (b) educated in a language other than English after age 10; (c) history of a neurological disorder (e.g., stroke or multiple sclerosis), TBI, learning disability, or psychiatric illness requiring hospitalization; (d) presence of any contraindication to MRI, (e) history of significant drug abuse other than alcohol; (f) presence of upper body injuries restricting the use of hands or arms; or (g) difficulties with eyesight.

We analyzed a subset of participants from the parent study that either (i) experienced an uncomplicated mTBI (*N* = 48; “mTBI group”), or (ii) a soft‐tissue or orthopedic injury without brain injury (*N* = 37; “OP controls”). Participants were included in the uncomplicated mTBI group if they presented to the emergency department following head trauma and met the following criteria: (a) loss of consciousness (LOC) from 0–30 min, (b) posttraumatic amnesia (PTA) of < 24 h, (c) Glasgow Coma Scale (GCS) score of 13 or 15, and (d) no trauma‐related intracranial abnormality on day‐of‐injury CT or 6–8 weeks structural MRI scan. Participants were included in the OP group if (a) they sustained a soft‐tissue or orthopedic injury below the neck; (b) there was no evidence of an altered state of consciousness as indicated by a reduction in GCS score, or presence of LOC, PTA, or posttraumatic confusion; and (c) there was no evidence of physical head trauma, whiplash, or cervical strain based on medical chart review (e.g., absence of lacerations/contusions to the head, absence of complaints of head, neck, or back pain). All participants whose lifetime consumption of alcohol or current alcohol consumption (operationalized as standard drinks per week) indicated a problematic alcohol use were excluded from this study. Additionally, mTBI participants and OP controls were matched for alcohol consumption insofar that they did not significantly differ in lifetime or current weekly alcohol consumption.

### 2.2 Study specific inclusion criteria

In addition to the previous inclusion and exclusion criteria, to be included in this study participants had to have a complete longitudinal set of T1 weighted longitudinal MRI data (first MRI exam 6 weeks after the injury, second MRI exam 12 months after injury) and their MRI data revealed no evidence of incidental findings at either of the two time‐points. Additionally, we ensured that the two groups did not statistically significantly differ on the following demographic variables: Age, sex, education, and alcohol consumption (Table 1). For more details of the participant selection process and exclusion criteria see Silverberg et al. (2016). A flow‐chart describing the selection process is available in the Supporting Information as Figure S1).

**TABLE 1:** Demographics

	mTBI	OP	Significance
Sample Size	48	37	
Age in Years	35 [10.6]	33 [9.7]	*p* = 0.39
Sex (male/female)	34 (71%) / 14 (29%)	25 (68%) / 12 (32%)	*p* = 0.81
**Ethnicity**			*p* = 0.22
Caucasian	37 (77%)	30 (81%)	
Asian‐Canadian	3 (6%)	3 (8%)	
Other	8 (17%)	4 (11%)	
Education in years	15.2 [2.6]	14.5 [2.1]	*p* = 0.20
Nonsmokers versus Smokers	34 (71%) /14 (29%)	23 (62%) / 14 (38%)	*p* = 0.49
Average number of drinks/week consumed in year before injury	5.8 [4.9]	4.3 [4.4]	*p* = 0.13
**Mechanism of Injury**			
Cyclist Accident	8 (17%)	8 (21%)	
Motor Vehicle Accident	5 (10%)	0 (0%)	
Assault	3 (6%)	3 (8%)	
Fall	6 (12%)	2 (5%)	
Pedestrian versus Car	4 (8%)	1 (4%)	
Sports Injury	2 (4%)	0 (0%)	
Other	20 (43%)	23 (62%)	
**Glasgow Coma Scale**			
15	15 (31%)		
14	29 (61%)		
13	4 (8%)		
**Loss of Consciousness Duration**			
None	2 (4%)		
Transient	11 (23%)		
<5 min	19 (40%)		
5–30 min	9 (19%)		
Could not be Determined	7 (14%)		
**Posttraumatic Amnesia Duration**			
<15 min	4 (8%)		
15–60 min	17 (36%)		
1–12 hours	24 (50%)		
12–24 hours	3 (6%)		

Note: Standard deviations are reported in squared brackets and percentages in brackets, mTBI stands for patients with uncomplicated mild traumatic brain injury, OP stands for orthopedic controls with either soft tissue or orthopedic injury below the neck.

All participants whose data were analyzed for this study gave written informed consent to participation in accordance with the Helsinki declaration and underwent procedures approved by the Research Ethics Board of the University of British Columbia pursuant to the Tri‐Council Policy Statement: Ethical Conduct for Research Involving Humans (TCPS2 2014), the International Conference on Harmonization Good Clinical Practice Guidelines (ICH‐GCP), and the requirements of the US Department of Health and Human Services for the Protection of Human Subjects 45CFR Part 46, sub‐part A.

### 2.3 MRI data

The first MRI exam took place between 6–8 weeks post injury and the second MRI exam 12 months post injury. All MRI data were acquired on the same Philips Achieva 3T scanner equipped with Dual Nova Gradients (maximum gradient strength 80 mT/m, max. slew rate 200 mT/m/s) and an eight‐channel head coil.

Although several structural MRI sequences were acquired during the 48 min MRI protocol, only the T1 weighted structural images were used for the results presented here: A T1 weighted TFE‐ SENSE sequence with TR = 8.161 ms, TE = 3.73 ms, Flip Angle = 99, FOV (ap/fh/lr) = 240 × 240 × 160 mm^3^, matrix size = 256 × 256, isotropic voxel size = 1 × 0.94 × 0.94 mm) 160 slices per volume.

### 2.4 VBM using the computational anatomical toolbox (CAT12)

In order to examine which brain regions showed significant GM changes during the first year after injury, we computed VBM using the default longitudinal preprocessing pipeline from the Computational Anatomic Toolbox (CAT12) (http://www.neuro.uni‐jena.de/cat/) that is implemented in the Statistical Parametric Mapping Toolbox (SPM12) (http://www.fil.ion.ucl.ac.uk/spm/) and run on MATLAB R2016a.

In addition to the standard processing steps of every VBM analysis (i.e., (a) segmentation of the T1 weighted image into the three tissue classes GM, WM, and cerebrospinal fluid [CSF], (b) normalization to a standard template like the 152 MNI, (c) modulation, and (d) smoothing) a longitudinal VBM pipeline has to consider that each subject has two or more time‐points that are dependent on each other. To account for this, the longitudinal pipeline of CAT12 starts with an inverse‐consistent realignment of all T1 weighted images of each subject that also includes bias correction between the different time‐points. Next, the average image of the realigned images is computed, and this average image is then segmented into GM and WM probability maps reflecting the probability of the respective tissue within each individual voxel. Subsequently, the spatial normalization parameters of the WM and GM probability maps of the average image are calculated using a DARTEL Normalization procedure (Ashburner, 2007). These normalization parameters are applied to the segmentations of both time‐points, before these are modulated and resampled to a 1.5 × 1.5 × 1.5 voxel size. In a final step, the GM and WM probability maps are smoothed using an isotropic Gaussian kernel (8 mm FWHM).

### 2.5 Statistical analyses VBM

The longitudinal pipeline of CAT12 uses the flexible factorial model of the SPM12 toolbox to meet the specific requirements of a longitudinal design. Factors were subject, group (mTBI group vs. OP controls), and timepoint (6 weeks vs. 1 year after injury). Total intracranial volume (TIV) was modeled as covariate of no interest (centering = overall mean) to control for differences of age, head‐size, and sex. An implicit mask with an absolute threshold of 0.1 was used to ensure that only GM voxels with intensities of 0.1 and higher were included in the analyses. The fit of the implicit mask with the individual smoothed GM/WM map of the participants was controlled by visual inspection.

Using the flexible factorial model of the SPM12 toolbox, the following statistical contrasts were computed: (a) Within‐change in the mTBI patients from 6 weeks to 12 months after injury, (b) within‐change in the OP controls from 6 weeks to 12 months after injury, (c) group difference between mTBI and OP participants in GM at 6 weeks, and (d) group differences between mTBI and OP participants in GM/WM at 12 months.

The nonparametric threshold‐free‐cluster‐enhancement (TFCE; permutation with 10,000 iterations) method in combination with the FWE correction (threshold *p* = 0.001) to control for multiple comparisons was used to detect voxel clusters indicating significant within‐subject change, between‐groups difference, and difference in amount of change. In contrast to other cluster‐based thresholding methods that do assume stationarity (= constant smoothness) of the data, TFCE does not make this assumption, provides better sensitivity as it is less affected by the smoothing kernel used, and does not require the user to arbitrarily specify an initial cluster‐forming threshold (Li et al., 2017; Salimi‐Khorshidi et al., 2009; Smith et al., 2009).

### 2.6 Post hoc analyses: How do the mTBI group's GM and WM changes relate to cognitive changes

To better understand the relevance of the GM and WM probability changes observed in the mTBI from 6 weeks to 1 year after the injury, we conducted a post hoc analysis to evaluate the association of these changes with attention performance. We used the standard score of attention from the Neuropsychological Assessment Battery (NAB, (White & Stern, 2003)) at both timepoints. The NAB consists of five domain‐specific modules: attention, language, memory, spatial, and executive functions. Although, mTBI patients tend to show at least temporary impairments in all five domains (Karr et al., 2014), we focused on attention for this study based on the right hemispheric predominance of the tissue changes observed in the mTBI group (see below for a detailed description of the regions showing tissue change in mTBI patients). Research has shown that attention is predominantly subserved by the right hemisphere (Corbetta et al., 2008), and the brain regions showing a decrease in GM probability in the mTBI participants (right superior and middle frontal gyris, right precentral gyrus with frontal eye field, and right anterior insula) are known to be involved in attention processes (Corbetta et al., 2008).

To relate the attention performance of the mTBI patients to their brain tissue change patterns, we binarized the thresholded SPM maps (*p* = 0.001 FWE corr.) and then used these binarized maps to extract the mean GM/WM probability values from the individual, total intercranial volume (TIV) corrected tissue maps at 6 weeks and 1 year after injury. We then computed the tissue change by subtracting the mean tissue probability value from 6 weeks from the value at 1 year. Next, the same was done with the individual standardized scores from the NAB attention module and the computed change in NAB attention score was then used to predict tissue change using a linear regression.

## 3 RESULTS

### 3.1 Results of the GM and WM analyses

Results of the two‐way repeated measures ANOVAs for group differences in global GM, WM, and CSF tissue volume (cm^3^) and average GM and WM probability at 6 weeks and 12 months

To compute the global volume of the three different tissue classes, CAT12 sums up the number of voxels constituting the GM, WM, and CSF probability maps without taking into account the probability values of the voxels.

Table 2 shows a summary of the global brain tissue (GMcm^3^, WMcm^3^, and CSFcm^3^) and mean GM and WM probability values for the two study groups at 6 weeks and 12 months. MTBI participants had minimally higher global GM volume and GM probability than controls, whereas controls had minimally higher global WM volume and WM probability values. To test whether these group differences in global tissue volume or tissue probability were significant at any of the two timepoints, we performed two‐way repeated measures ANOVAs for GM, WM, and CSF and GM and WM probability after scaling the tissue values with the TIV to correct for differences in head size. The two‐way repeated measures ANOVAs revealed no significant group and time effects.

**TABLE 2:** General overview: Global GM, WM and CSF volume, and global GM and WM probability of mTBI patients and controls at T1 and T2

	mTBI		Controls	
	6 Weeks	1 Year	6 Weeks	1 Year
**GM Volume in cm^3^ (TIVcorr)**	437.11 (26,21)	436.49 (23.50)	434.61 (25.71)	434.70 (28.48)
**WM Volume in cm^3^ (TIVcorr)**	337.72 (16.74)	337.02 (17.85)	342.96 (18.33)	343.73 (18.43)
**CSF Volume in cm^3^ (TIVcorr)**	225.16 (27.96)	226.49 (26.61)	222.44 (27.50)	221.58 (31.45)
**Degree of Atrophy**	1.29 (0.05)	1.29 (0.04)	1.29 (0.04)	1.29 (0.05)
**GM Probability (TIVcorr)**	0.329 (0.016)	0.329 (0.016)	0.328 (0.016)	0.328 (0.018)
**WM Probability (TIVcorr)**	0.371 (0.017)	0.372 (0.018)	0.377 (0.018)	0.377 (0.019)

Note: TIV stands for total intracranial volume, the degree of atrophy was computed by dividing TIV by the sum of GM and WM volume, a higher value means higher atrophy, the standard deviation values are listed in brackets. The table shows that the global GM and WM parameter were quite stable for both groups from 6 weeks to 1 year after injury. MTBI patients had at both timepoints higher GM volumes and GM probability values than controls (all comparisons *p* > 0.05), but lower WM volumes and WM probability than controls (all comparisons *p* > 0.05).

### 3.2 Results of the VBM: Between group differences in GM and WM at 6 weeks and at 12 months

We used two‐sample *t*‐tests to test for differences in GM/WM probability between the groups at 6 weeks and at 12 months after injury. Using the TFCE method with subsequent FWE correction, we found no significant clusters indicating a difference in voxel‐wise GM/WM probability between the mTBI group and the controls either at 6 weeks or at 1 year after injury. These findings remained even after lowering the threshold to a very lenient level of *p* = 0.001 uncorrected.

### 3.3 Results of the VBM: Within‐subject reorganization from 6 weeks to 12 months

We tested for significant within‐subject GM/WM change on voxel‐level using the flexible factorial model as implemented in SPM12 while controlling for TIV and age. Only the within‐subject result for the mTBI group was significant at *p* < 0.001 using the TFCE method in combination with FWE correction.

#### 3.3.1 Decrease in GM probability in the mTBI participants from 6 weeks to 1 year

**FIGURE 1:**
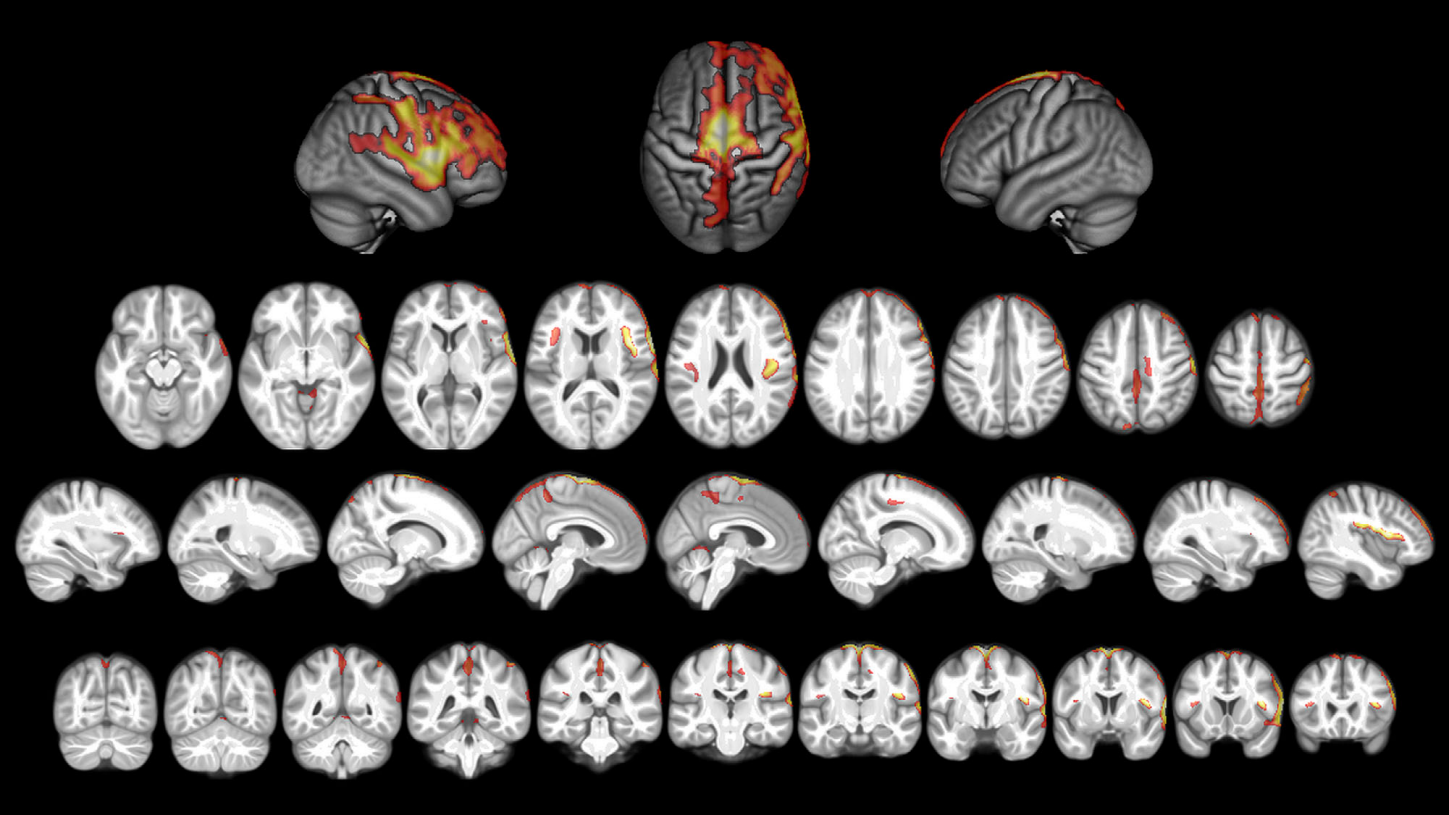
Voxel‐wise gray matter (GM_ probability reduction in mild traumatic brain injury (mTBI) participants from 6 weeks to 1 year after injury (*p* = 0.001 FWE corr. TFCE). Red color highlights brain regions with GM probability reduction at *p* = 0.001 FWE corr. (threshold‐free‐cluster‐enhancement [TFCE]), orange–yellow color highlights brain regions with *p* < 0.001 FWE corr. Brain regions highest degree of significant GM probability reduction such as the bilateral precentral gyrus, bilateral supplementary motor cortex, right rolandic operculum, and right anterior insula have the are colored yellow

The mTBI patients showed a significant decrease of GM probability in nine clusters extending over 14,000 voxels predominantly on the right hemisphere (see Figure 1 and Table S2 with cluster sizes and peak coordinates in the Supporting Information). To identify the brain regions showing GM reduction, the automated anatomical labeling atlas (AAL3; Rolls et al., 2020) was used. The brain regions showing the highest degree of GM probability reduction on the right hemisphere were the rolandic operculum (23.04% = percentage of the entire brain region showing GM probability reduction), inferior frontal gyrus (pars opercularis 21.52% and pars triangularis 14.56%), supplemental motor area (13.13%), paracentral lobe (11.77%), superior temporal pole (10.43%), middle frontal gyrus (9.35%), superior temporal gyrus (8.42%), postcentral gyrus (8%), supramarginal gyrus (7.59), precentral gyrus (7.42%), and insula (6.44%). On the left hemisphere, the supplemental motor area (19.92%), paracentral lobe (16.29), precuneus (11.83%), vermis 4 and 5 of the cerebellum (7.09%), medial superior frontal gyrus (6.63%), and middle part of the cingulate cortex (5.76%) showed GM probability reduction 1 year after injury.

#### 3.3.2 Changes in WM probability in mTBI patients from 6 weeks to 1 year after injury

**FIGURE 2:**
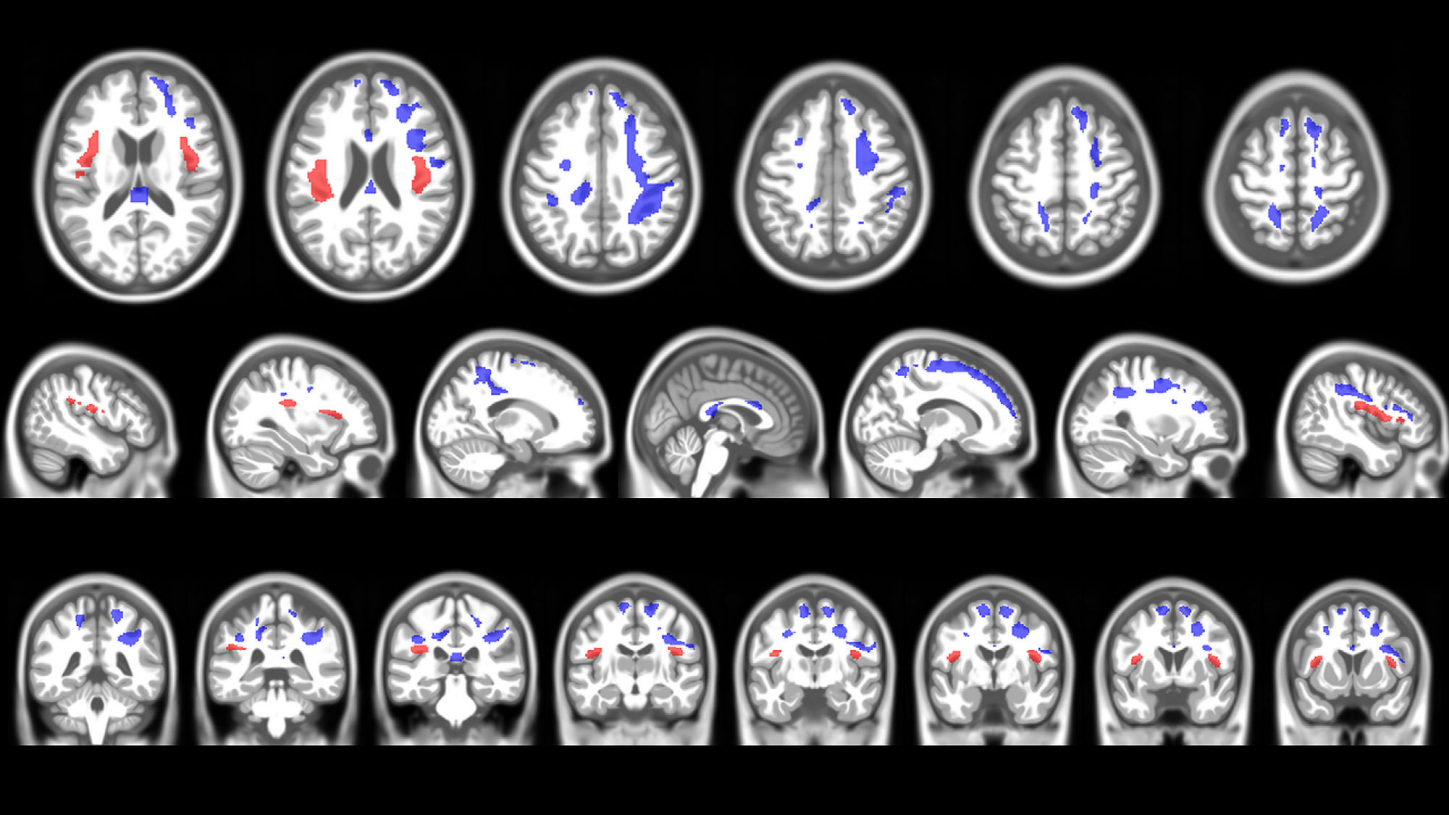
Voxel‐wise white matter (WM) probability increase and decrease in mTBI participants from 6 weeks to 1 year after injury (*p* = 0.001 FWE corr. TFCE). Red‐colored clusters show brain regions with significant voxel‐wise WM probability increase and blue colors show brain regions with significant voxel‐wise decrease in WM probability in the mTBI group from 6 weeks to 1 year after injury

The mTBI patients showed WM probability decrease in 12 clusters encompassing 13,180 voxels predominantly located in the right hemisphere, and to a lesser degree, bilateral WM probability increase in two clusters covering 3,687 voxels (see Figure 2 and Tables S3 and S4). The WM tract atlas of (Yeh et al., 2018) was used to map the clusters with WM probability changes to discrete WM tracts.

In the right hemisphere, the following tracts showed a decrease in WM probability: superior longitudinal fasciculus (33.39%), frontal aslant tract (30.28%), frontopontine (23.51%), and parietopontine (19.47%) tracts, arcuate fasciculus (18.83%), cortico‐striatal pathway (17.95%), cortico‐spinal tract (16.62%), capsula extrema (16.24%), corticothalamic pathway (15.83%), and middle longitudinal fasciculus (10.28%), and 16.88% of the short association U fibers which connect adjacent gyri and are located in WM near the GM‐WM boundary. Additionally, 7.09% of the bilateral corpus callosum showed WM probability reduction and, in the left‐brain side, mainly parietopontine tract (5.61%) and corticospinal tract (5.24%) were affected by WM probability decrease. In contrast, WM probability increase was mainly observed in the left‐brain hemisphere in the following tracts: arcuate fasciculus (6.47%), capsula extrema (3.59%), and in 3.52% of the U fibers.

**FIGURE 3:**
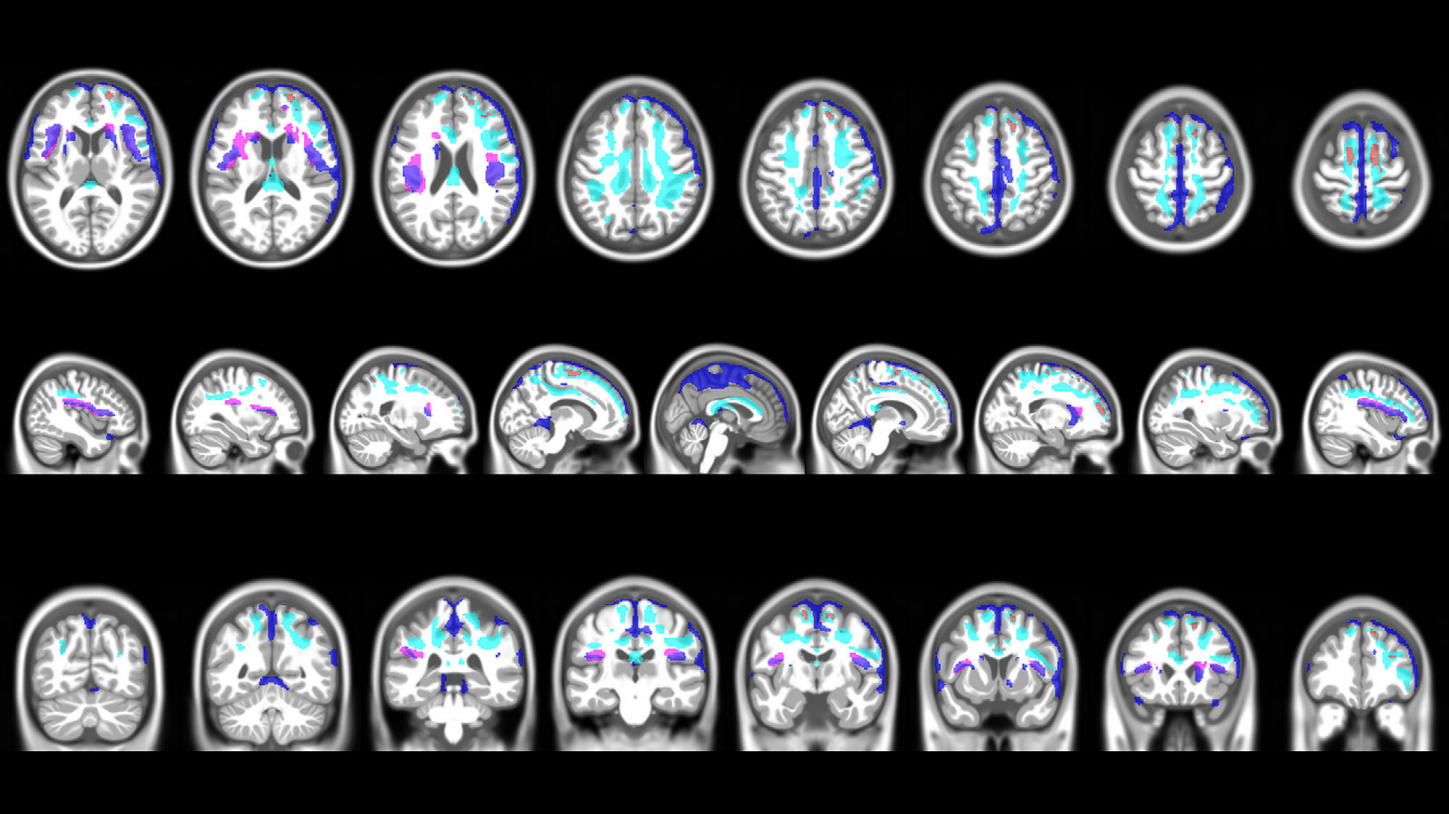
Combined voxel‐wise GM and WM changes in mTBI participants. The mTBI participants GM and WM probability changes from 6 weeks to 1 year are overlayed on the same brain template to illustrate the spatial relationship of the tissue changes. To allow for an easier comparison of the voxel‐wise change pattern, the statistical threshold was lowered to 0.01 FWE corr. (TFCE). Dark blue color indicates brain regions with a significant GM decrease and red color highlights brain regions with a small but significant GM increase (see row 1, the first three axial slices, right superior frontal gyrus). Cyan color indicates brain regions with a WM decrease and violet color highlights brain regions with a WM increase

Figure 3 shows the mTBI participants’ WM and GM changes in the relation to each other. The amount of GM and WM probability reduction was with 14,000 voxels (GM) and 13,180 voxels (WM) almost the same, and both reduction patterns were located predominantly in the right hemisphere. In contrast, the amount of WM increase was much smaller (approximately three times smaller) than the GM/WM reduction. A striking feature of the mTBI groups’ GM probability decrease pattern was that it followed closely the GM‐CSF border and the deeper layers of the cortical ribbon did not seem to be affected. The WM probability increase pattern, on the other hand, was located along the GM‐WM border opposite the GM probability reduction pattern. We had used implicit masking with an absolute threshold of 0.1 to compute the voxel‐wise statistics. To exclude the possibility of a masking artefact, we repeated the VBM analysis for GM and WM with an absolute threshold of 0.2. Even then we saw still the same GM/WM probability reduction patterns following the GM‐CSF border, respectively the opposite GM‐WM border, after lowering the statistical threshold to *p *= 0.05 FWE corrected.

### 3.4 Results of the post hoc tests

**FIGURE 4:**
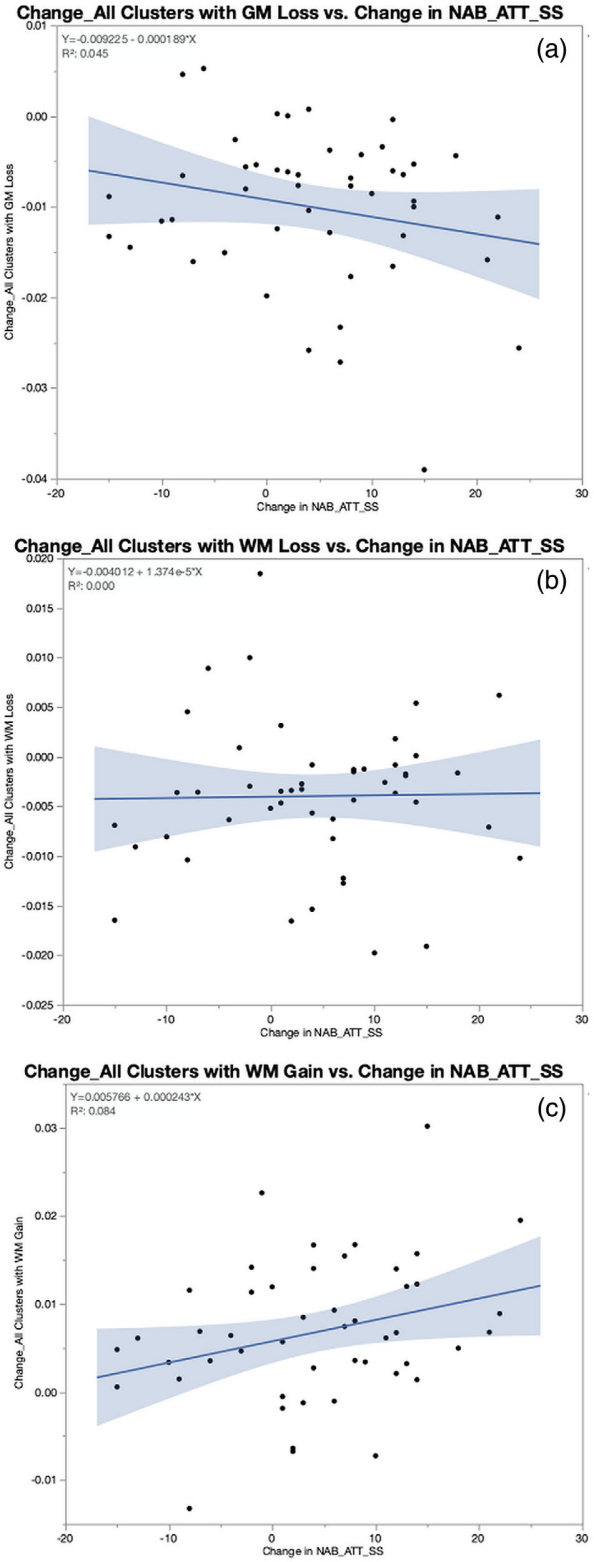
Results of the regression analyses: Improvement in attention predicts WM increase in mTBI participants but not GM and WM decrease. (a) shows the scatterplot for the regression change in clusters with GM decrease during the period from 6 weeks to 1 year after injury vs improvement in attention (operationalized as neuropsychological assessment battery [NAB] attention module standard score (SS) at 1 year after injury minus NAB attention module SS at 6 weeks after injury. (b) shows the scatterplot for the regression change in clusters with WM decrease during the period from 6 weeks to 1 year after injury vs improvement in attention and Figure 4C shows the scatterplot for change in clusters with WM increase during the period from 6 weeks to 1 year after injury versus improvement in attention. Only WM increase (*R* ^2^ = 0.084; *F*(1,46) = 4.2291; *p* = 0.0454), but neither GM reduction (*R* ^2^ = 0.045; *F*(1,46) = 2.1919; *p* = 0.1456) or WM reduction (*R* ^2^ = 0.00032; *F*(1,46) = 0.0147; *p* = 0.9041) was significantly predicted by the mTBI patients’ improvement in NAB attention module

The standard score of the NAB Attention module was used to understand the relationship between the tissue changes observed in the mTBI group and cognitive outcome 1 year after injury. The comparison of OP controls and mTBI patients showed that the two groups did not differ significantly from each other in the NAB Attention Module at either of the two time points (mTBI patients: mean = 105.59, SD = 12.96; OP controls: mean = 104.40, SD = 15.29; *t*(70.4) = 0.375; *p* = 0.7081 at 6 weeks after injury; mTBI patients: mean = 109.87, SD = 14.62; OP controls: mean = 105.73, SD = 14.95; *t*(76.71) = 1.279; *p* = 0.2047 at 1 year after injury). However, the mTBI patients’ attention performance improved significantly from 6 weeks to 1 year (*t*(47) = 3.104; *p* = 0.0032), whereas the OP controls’ attention performance did not (*t*(36) = 1.001; *p* = 0.3234). The regression analyses revealed that only WM increase (*R* ^2^ = 0.084; *F*(1,46) = 4.2291; *p* = 0.0454), but neither GM reduction (*R* ^2^ = 0.045; *F*(1,46) = 2.1919; *p* = 0.1456) or WM reduction (*R* ^2^ = 0.00032; *F*(1,46) = 0.0147; *p* = 0.9041) was significantly predicted by the mTBI patients’ improvement in NAB Attention Module (see Figure 4).

## 4 DISCUSSION

The aim of this study was to investigate whether we can detect regional changes in GM and WM following uncomplicated, mild traumatic brain injury in the first year after injury. To that end, we conducted a VBM analysis in 48 mTBI patients and 37 OP controls at 6 weeks and 1 year after injury using a longitudinal preprocessing pipeline optimized to detect subtle changes in combination with a nonparametric cluster‐wise statistic on voxel‐level.

We did not observe any significant group differences in global GM and WM volume or in GM and WM probability at each of the two timepoints. This finding was not completely unexpected, given that mTBI patients had minimally higher global GM volume and average GM probability values and only slightly lower global WM volume and average WM probability than controls at both timepoints (see Table 2). However, although the mTBI patients did not significantly differ from the OP controls at any time, they still underwent significant change (decrease and increase) in GM and WM probability during the first year after injury. The GM probability decreases were predominantly located in the right anterior part of the brain (rolandic operculum, precentral gyrus, middle frontal gyrus, supplementary motor area, insula, superior temporal pole) following the outer GM border to the CSF with corresponding WM probability decreases located in parts of superior fasciculus longitudinalis, arcuate fasciculus, and cortical‐pontine tracts. On the other hand, the mTBI patients also showed bilateral WM probability increase, albeit to a smaller degree than the decrease (13,1180 voxels vs 3,687 voxels), in WM regions below rolandic operculum and insula (arcuate fasciculus, capsula extrema, and the U fibers near insula and rolandic operculum).

There are two possible explanations for this distinct GM/WM probability change patterns in the mTBI group: it may just be an artefact, or it has a true biological cause. We start by excluding the possibility of our findings being artificial. A masking artefact could theoretically generate the kind of change pattern seen in mTBI patients. We addressed this possibility by using a more rigorous masking approach (implicit mask with an absolute threshold of 0.2 instead of an absolute threshold of 0.1 that was used for the analyses presented here) and still found the same GM probability change pattern. Not an artefact in the proper sense, but more a factor that has to be kept in mind when interpreting VBM results is the fact that we used “modulated” tissue maps. Modulation means that a voxel's intensity not just provides information about the voxel's probability to belong to one of the tissue categories but also if the voxel had to be expanded or contracted during the coregistration onto the population average. Expansion is typically interpreted as evidence for a loss of tissue volume, contraction as tissue volume gain. Because they incorporate intensity and volume information, modulated tissue maps are considered to be more sensitive than nonmodulated maps. However, the downside of this increased sensitivity is that it is no longer possible to easily distinguish if a local change in the probability map is caused by a reduction of tissue volume resulting in an expansion during the normalization step or a change of a voxel's probability to be categorized as GM respectively WM or a combination of the two. The distribution of the change patterns along the tissue borders in the mTBI group however suggests that the change is more likely caused by an intensity change than by a volume reduction.

An alternative explanation—and an indicator of an underlying biological cause—is that the change pattern in the mTBI subjects is caused by a diffuse intensity shift of the T1 weighted signal mostly in the frontal cortex and to a lesser degree also in the sensorimotor cortex from 6 weeks to 12 months. Palacios and colleagues (2013) showed that traumatic brain injury is associated with a reduction of the T1 weighted signal intensity contrast in almost all brain regions. Contrast reduction leads to a blurring of the WM‐GM boundary that affects the accuracy of the algorithms used for the segmentation of the T1 weighted image into the different tissue classes. The determination of the WM‐GM boundary and the GM‐CSF boundary are crucial steps during segmentation. CAT12 and other software suites like SPM12 or FSL use the intensity of each voxel together with spatial information from tissue priors to estimate each voxels probability to belong to white or GM and generate tissue probability maps. Therefore, they are able to capture gray/white intensity changes in contrast to software suites using a binary approach, as for example Freesurfer (Chung et al., 2017).

It is well known that the intensity of the T1 weighted image is susceptible to other MRI parameters like proton density or water content, region‐specific degrees of myelination, and iron deposition (Bansal et al., 2013; Lorio et al., 2016). Mild TBI can result in a change of the brain's water content (caused by local edema as a result of the impact at the accident), or in abnormally high accumulation of iron (caused by microscopic brain damage, oxidative stress injury, increased blood‐brain permeability, or hemoglobin degradation products, (see Lu et al., 2015; Nisenbaum et al., 2014; Raz et al., 2011), or in changes of degrees of myelination.

Which of the above listed biological causes can explain best the GM probability changes on the WM‐GM and the GM‐CSF boundaries we observed in the mTBI patients? The locations of edemas resulting from the impact when the brain collided with the skull during the accident are highly individual and would therefore not show up as a clearly defined pattern in a group analysis. Furthermore, an edema would cause a local decrease in signal intensity at first before the signal intensity would gradually return to its original height as the brain recovers, but for large parts of the GM and WM we observed the opposite shift in brain signal intensity. Abnormally high iron accumulations after mTBI are usually found in subcortical structures such as the thalamus and the basal ganglia (Lu et al., 2015; Raz et al., 2011), but thalamus and basal ganglia were not found affected by GM probability decrease in the mTBI group at *p* = 0.001 FWE corr.

After excluding the first two possible explanations, the only other explanation left is a change in the degree of myelination. The term myelination is commonly associated with the oligodendrocytes in the WM that insulate the long‐range axons to increase the conduction velocity of the action potential and to reduce the energetic cost. We analyzed T1 weighted images which do have only limited information for understanding changes in WM, but myelin is also found in GM. Sprooten et al. (2019) demonstrated that the highest amount of intracortical myelin can be observed in the deep layers of the GM near the WM‐GM border, and Micheva et al. (2016) showed that a substantial portion of the intracortical myelin insulates axons of GABA‐ergic interneurons. In the context of mTBI, Vascak and colleagues (2018) recently showed in a mice model of mTBI that mTBI can result in diffuse axonal injury located in the interneurons of the intracortical myelin in the GM.

Two key publications (King et al., 2016; Ghajari et al., 2017) support the assumption that the tissue changes found in the mTBI group could represent a joint phenomenon affecting both GM and WM. For example, increased mean cortical curvature in GM is commonly interpreted as a biomarker for WM atrophy and King and colleagues (2016) used this GM parameter with the goal to detect progressive tissue degeneration in veterans with mTBI. They found a pattern of increased mean cortical curvature that showed interestingly the same right‐dominant asymmetry that we observed (King et al., 2016). Furthermore, the medial frontal brain regions highlighted by our VBM findings are in line with the contour patterns of mechanical strain and strain‐rate that were found by Ghajari et al. (2017) when they simulated the injury biomechanics that act upon the brain during a motor vehicle accident, a sport accident (American football), or a fall. Ghajari and colleagues (2017) also showed that the brain regions that were found to be exposed to the highest values of strain and strain‐rate in the simulation were also associated with significantly reduced fractional anisotropy values in the WM nearest to the WM‐GM boundary, particularly in the sulci, in a group of patients who had suffered one of the three modeled brain injuries.

Based on the arguments above, we suggest that the tissue change patterns observed in the mTBI patients during the first year after injury represent a neural reorganization process of the brain than actual volume loss. This assumption is supported by the fact that the improvement in attention performance in the mTBI patients was significantly predicted by their WM probability increase in brain regions involved in attentional processes during the same period. Although neither the GM nor the WM probability decreases were significantly related to the mTBI group's improvement in attention performance, it is interesting that GM decrease was inversely related with attention improvement. MTBI patients with higher GM probability decrease tended to improve more than mTBI patients with an increase in GM probability (see Figure 4a). Combined with the fact that mTBI patients had higher average GM probability values than controls at both timepoints, this suggests that the persistent higher GM probability values in mTBI patients with less improvement in attention could have been the result of a maladaptive process. This speculation is in line with a similar finding of Dall'Acqua et al. (2017) who found that mTBI patients with exaggerated cortical thickening showed a worsening cognitive performance during the first year after injury, while mTBI patients with cortical thinning in the same range than the healthy controls improved cognitively.

### 4.1 Limitations

Our control group consisted of another patient sample, that is., patients treated for soft‐tissue or orthopedic injury at the emergency department of Vancouver General Hospital during the period when the data of the mTBI patients were collected. OP patients are the ideal control population to investigate the consequences of mTBI because they share many premorbid characteristics and injury‐related experiences with the mTBI patients that are not equally shared with the population of healthy controls. The use of OP patients as control group is an elegant way to control for these potential confounds and to make sure that all findings are related to the specific consequences of a traumatic brain injury only and not influenced by mTBI unspecific factors like being in pain or being hospitalized for some time. However, the lack of a healthy control group made it difficult to understand the exact nature of the changes observed in the OP controls. We cannot be sure whether these changes reflect a normal aging process, or a recovery from the injury and immobilization because it has been shown that immobilization can result in plastic GM and WM changes (Langer et al., 2012), or a mixture of both.

## 5 CONCLUSION

Our study participants had a mean age of 34.5 of years and, therefore, were still in an age‐range where one would expect to find only minimal age‐related GM reduction within 1 year (Sowell et al., 2003). The lack of significant longitudinal GM changes in the OP controls confirmed this expectation. In contrast, the VBM showed that the mTBI group had undergone significant GM and WM probability decreases along the tissue borders of the right rolandic operculum, right insula, right precentral gyrus, right middle frontal gyrus, and right superior temporal pole in combination with WM probability increase predominantly in the WM underlying the left rolandic operculum and left insula 12 months after injury. Interestingly, we did not detect a group difference in GM or WM probability between mTBI patients and controls at either timepoint despite the significant changes in the mTBI group. This suggest that the GM changes observed in the mTBI patients may rather be qualitative (= intensity change of the T1 weighted image) than quantitative (= volume loss). In combination with the finding that the mTBI group showed an improved attention performance at 1 year after injury, we interpret the VBM findings as reorganization caused by subtle microstructural changes in myelination in GM and WM. However, confirmation from studies on large and well characterized samples of patients with mild uncomplicated TBI, including orthopedic controls as well as healthy controls for comparison, combining other MRI modalities, for example, DTI or myelin mapping, with T1 weighted MRI data, and with a well‐standardized protocol and rigorous quality control in place is needed.

### PEER REVIEW

The peer review history for this article is available at https://publons.com/publon/10.1002/brb3.2410

## Supporting information

Supporting Information

## Data Availability

The data that support the findings of this study are available from the principal investigator of the study, Rael T Lange (rlange@dvbic.org) upon reasonable request.
