# Supplementary material for: Longitudinal changes in brain parenchyma due to mild traumatic brain injury during the first year after injury
Source: Brain Behav. 2021 Oct 28;11(12):e2410. doi: 10.1002/brb3.2410 (PMC8671787; doi:10.1002/brb3.2410)
Supplement: Supplementary file 1 — Supporting Information [file BRB3-11-e2410-s001.docx]

**Supplement**

**Figure S1 Flowchart illustrating the selection process and exclusion criteria**

Could not be contacted (n = 663)

Potentially eligible after chart review

(n = 1,931)

TBI = 1,158; OPC = 773

Telephone screen to determine eligibility and consent ( n = 1,268)

TBI = 762; OPC 506

**Excluded ( n = 1,030)**

Too busy/no time (n = 180)

Not interested (n = 377)

Diagnosis unclear (n = 99)

Prior neurological condition (n =25)

Illicit drug abuse history (n = 58)

Psychiatric history (n = 9)

Cancer history (n = 13)

From out of town (n = 31)

ESL (n = 128)

Contradiction to MRI (n = 27)

Medically unstable ( n = 54)

Learning Disability (n = 29)

**Excluded (n = 19)**

Not eligible for follow-up. The anniversary of the injury data was <12 months before the study ended

**Excluded from TBI group (n = 9)**

Severe TBI Glasgow Coma Scale < 12 or posttraumatic amnesia > week

Consented to study and completed baseline testing

(n = 238)

TBI = 155; OPC 83

Completed baseline testing and eligible for follow-up

(n = 219)

TBI = 144; OPC = 75

Mild-moderate TBI (n = 135)

**Excluded from OPC group (n = 21)**

Incidental findings (n = 4); One or more white matter hyperintensities on MRI ( n= 17)

OPC

(n =75)

**Excluded from OPC group (n = 14)**

Weekly alcohol consumption over

20 standard drinks

**Excluded from TBI group (n = 52)**

Fulfilling the criteria for moderate or complicated mild TBI

Completed

Follow-up

(n = 91)

Mild uncomplicated TBI (n = 49)

**Excluded from mTBI group (n = 1)**

Weekly alcohol consumption over

20 standard drinks

Usable for MRI comparison

(n = 54)

Included in this analysis

(n = 37)

Included in this analysis

(n = 48)

**Table S2 Results of the VBM: GM probability decrease in the mTBI patients from six weeks to one year after injury (height threshold p < 0.001 FWE; extend threshold k = 0 voxels)**

|  |  |  |  |  |  |  |  |
| --- | --- | --- | --- | --- | --- | --- | --- |
| **Combined Peak Cluster Level** | | | |  |  |  | **Location of the Peak Voxel** |
| **k** | **p(FWE-corr)** | **p(FDR-corr)** | **TFCE** | **x** | **y** | **z {mm}** |  |
| **5362** | **0** | **0.002** | **4429.15** | **-2** | **-6** | **74** | **Left Posterior Part of the Medial Superior Frontal Cortex** |
|  | 0 | 0.002 | 4330 | 0 | -18 | 78 | Right Paracentral Lobe |
|  | 0 | 0.002 | 4087.4 | -15 | -14 | 80 | Left Precentral Gyrus |
| **1259** | **0** | **0.002** | **4369.9** | **44** | **-3** | **20** | **Right Rolandic Operculum** |
|  | 0 | 0.002 | 4205.19 | 40 | 9 | 14 | Right Inferior Frontal Gyrus/Pars Opercularis |
|  | 0 | 0.002 | 3902.66 | 38 | 21 | 12 | Right Inferior Frontal Gyrus/Pars Triangularis |
| **6749** | **0** | **0.002** | **4357.14** | **63** | **10** | **10** | **Right Rolandic Operculum** |
|  | 0 | 0.002 | 4224.12 | 64 | 2 | 6 | Right Rolandic Operculum |
|  | 0 | 0.002 | 3982.72 | 63 | 8 | -4 | Right Temporal Pole |
| **274** | **0** | **0.002** | **3129.55** | **-36** | **20** | **14** | **Left Inferior Frontal Gyrus/Pars Triangularis** |
|  | 0.001 | 0.002 | 2918.77 | -36 | 6 | 16 | Left Rolandic Operculum |
|  | 0.001 | 0.002 | 2826.03 | -39 | -2 | 20 | Left Insula |
| **1** | **0.001** | **0.002** | **2813.42** | **-8** | **-26** | **81** | **Left Paracentral Lobule** |
| **200** | **0.001** | **0.002** | **2806.86** | **6** | **-42** | **-4** | **Right Cerebellar Vermis/Lobule IV** |
|  | 0.001 | 0.002 | 2769.42 | 3 | -58 | -3 | Right Cerebellar Vermis/Lobule V |
|  | 0.001 | 0.003 | 2691.68 | -6 | -56 | -2 | Left Cerebellum/Lobule V |
| **137** | **0.001** | **0.002** | **2794.67** | **-44** | **-16** | **22** | **Left Rolandic Operculum** |
|  | 0.001 | 0.002 | 2778.51 | -34 | -28 | 24 | Left Posterior Insula |
| **2** | **0.001** | **0.002** | **2666.38** | **10** | **-30** | **81** | **Right Paracentral Lobule** |
| **16** | **0.001** | **0.002** | **2661.96** | **-4** | **-42** | **-6** | **Left Cerebellum/Lobule IV** |
|  |  |  |  |  |  |  |  |
| Degrees of freedom = [1.0, 82.0] | | | |  |  |  | Voxel size = 1.5 1.5 1.5 mm (resel = 494.07 voxels) |
| FWHM = 11.5, 12.3, 11.8 {mm}; 7.7 8.2 7.9 {voxels} | | | |  |  |  | Permutations = 10,000 |
| Volume = 1484179 = 439762 voxels = 795.4 resels | | | |  |  |  |  |

**Table S2:** 9 clusters showed significant GM probability decrease in the mTBI patients (bold letters, with three local maxima more than 8 mm apart in standard letters). The first column on the left side shows the number of voxels (kE) constituting the clusters, the second column lists the FWE corrected p values for the clusters followed by the FDR corrected p values for the corresponding peak voxels and the corresponding TFCE value. The third column lists the MNI coordinates of the peak voxels, and the fourth column lists the location of the peak voxels in the brain.

**Table S3 Results of the VBM: WM probability decrease in the mTBI patients from six weeks to one year after injury (height threshold p < 0.001 FWE; extend threshold k = 0 voxels)**

|  | | | |  |  |  |  |
| --- | --- | --- | --- | --- | --- | --- | --- |
| **Combined Peak Cluster Level** | | | |  |  |  | **Location of the Peak Voxel** |
| **k** | **p(FWE-corr)** | **p(FDR-corr)** | **TFCE** | **x** | **y** | **z {mm}** |  |
| **9217** | **0** | **0.001** | **3912.15** | **34** | **-39** | **33** | **Right Superior Fasciculus Longitudinalis** |
|  | 0 | 0.001 | 3573.5 | 22 | -8 | 44 | Right Superior Corona Radiata |
|  | 0 | 0.001 | 2975.06 | 32 | -16 | 34 | Right Superior Fasciculus Longitudinalis |
| **880** | **0** | **0.001** | **2674.03** | **-9** | **-6** | **63** | **White Matter below Left Superior Frontal Gyrus** |
|  | 0 | 0.001 | 2521.29 | -10 | 3 | 66 | White Matter below Left Superior Frontal Gyrus |
|  | 0 | 0.001 | 2494.92 | -10 | -14 | 69 | White Matter below Left Superior Frontal Gyrus |
| **772** | **0** | **0.001** | **2458.43** | **36** | **18** | **21** | **Right Superior Corona Radiata** |
|  | 0 | 0.001 | 2239.38 | 33 | 6 | 26 | White Matter below Right Middle Frontal Gyrus |
|  | 0.001 | 0.001 | 2088.83 | 40 | 28 | 16 | White Matter below Right Middle Frontal Gyrus |
| **1018** | **0** | **0.001** | **2321.23** | **-16** | **-48** | **56** | **White Matter below Left Superior Parietal Lobule** |
|  | 0.001 | 0.001 | 2058.59 | -16 | -54 | 46 | White Matter below Left Superior Parietal Lobule |
|  | 0.001 | 0.002 | 2042.42 | -22 | -33 | 38 | Left Posterior Corona Radiata |
| **406** | **0** | **0.001** | **2245.54** | **3** | **-34** | **18** | **Right Corpus Callosum** |
|  | 0 | 0.001 | 2145.29 | 2 | -24 | 24 | Right Corpus Callosum |
| **275** | **0** | **0.001** | **2170.36** | **-36** | **-33** | **33** | **Left Superior Fasciculus Longitudinalis** |
| **148** | **0** | **0.001** | **2160.97** | **0** | **14** | **24** | **Left Corpus Callosum** |
| **156** | **0.001** | **0.001** | **1994.69** | **-9** | **58** | **24** | **White Matter below Left Superior Frontal Gyrus** |
|  | 0.001 | 0.001 | 1989.01 | -16 | 52 | 27 | White Matter below Left Superior Frontal Gyrus |
|  | 0.001 | 0.001 | 1948.47 | -9 | 56 | 32 | White Matter below Left Superior Frontal Gyrus |
| **155** | **0.001** | **0.001** | **1968.4** | **-26** | **-8** | **39** | **Left Superior Corona Radiata** |
|  | 0.001 | 0.002 | 1911.98 | -18 | -6 | 42 | Left Superior Corona Radiata |
| **83** | **0.001** | **0.001** | **1966.8** | **-27** | **10** | **44** | **Left Superior Corona Radiata** |
| **68** | **0.001** | **0.001** | **1937.32** | **-6** | **24** | **58** | **White Matter below Left Superior Frontal Gyrus** |
|  | 0.001 | 0.001 | 1918.17 | -10 | 16 | 57 | White Matter below Left Superior Frontal Gyrus |
| **2** | **0.001** | **0.001** | **1914.18** | **42** | **38** | **26** | **White Matter below Right Middle Frontal Gyrus** |
|  |  |  |  |  |  |  |  |
| Degrees of freedom = [1.0 82.0] | | | |  |  |  | Voxel size 1.5 1.5 1.5 mm; (resel = 461.97 voxels) |
| FHWM = 11.3 12.3 11.3 {mm} 7.6 8.1 7.5 {voxels} | | | |  |  |  | Permutations = 10000 |
| Volume = 989702 = 293245 voxels = 556.2 resels | | | |  |  |  |  |

**Table S3:** 12 clusters showed significant WM probability decrease in the mTBI patients (bold letters, with three local maxima more than 8 mm apart in standard letters). The first column on the left side shows the number of voxels (kE) constituting the clusters, the second column lists the FWE corrected p values for the clusters followed by the FDR corrected p values for the corresponding peak voxels and the corresponding TFCE value. The third column lists the MNI coordinates of the peak voxels, and the fourth column lists the location of the peak voxels in the brain.

**Table S4 Results of the VBM: WM probability increase in the mTBI patients from six weeks to one year after injury (height threshold p < 0.001 FWE; extend threshold k = 0 voxels)**

|  | | | |  |  |  |  |
| --- | --- | --- | --- | --- | --- | --- | --- |
| **Combined Peak Cluster Level** | | | |  |  |  | **Location of the Peak Voxel** |
| **k** | **p(FWE-corr)** | **p(FDR-corr)** | **TFCE** | **x** | **y** | **z {mm}** |  |
| **1686** | **0** | **0.001** | **4072.77** | **42** | **-3** | **16** | **Right Superior Fasciculus Longitudinalis** |
|  | 0 | 0.001 | 3744.54 | 40 | 10 | 14 | White Matter below Right Middle Frontal Gyrus |
|  | 0 | 0.001 | 3381.31 | 38 | 21 | 10 | Right Anterior Corona Radiata |
| **2001** | **0** | **0.001** | **3126.79** | **-36** | **21** | **14** | **White Matter below Left Middle Frontal Gyrus** |
|  | 0 | 0.001 | 3113.51 | -38 | 8 | 14 | Left Capsula Externa |
|  | 0 | 0.001 | 3044.06 | -39 | -2 | 20 | White Matter below Left Precentral Gyrus |
|  |  |  |  |  |  |  |  |
| Degrees of freedom = [1.0 82.0] | | | |  |  |  | Voxel size 1.5 1.5 1.5 mm; (resel = 461.97 voxels) |
| FHWM = 11.3 12.3 11.3 {mm} 7.6 8.1 7.5 {voxels} | | | |  |  |  | Permutations = 10000 |
| Volume = 989702 = 293245 voxels = 556.2 resels | | | |  |  |  |  |

**Table S:** 2 clusters showed significant WM probability increase in the mTBI patients (bold letters, with three local maxima more than 8 mm apart in standard letters). The first column on the left side shows the number of voxels (kE) constituting the clusters, the second column lists the FWE corrected p values for the clusters followed by the FDR corrected p values for the corresponding peak voxels and the corresponding TFCE value. The third column lists the MNI coordinates of the peak voxels, and the fourth column lists the location of the peak voxels in the brain.
